# Effect of the duty cycle of the ultrasonic processor on the efficiency of extraction of phenolic compounds from *Sorbus intermedia*

**DOI:** 10.1038/s41598-022-12244-y

**Published:** 2022-05-18

**Authors:** Zbigniew Kobus, Monika Krzywicka, Agnieszka Starek-Wójcicka, Agnieszka Sagan

**Affiliations:** 1 Department of Technology Fundamentals, University of Life Sciences in Lublin, Głęboka 28, 20-612 Lublin, Poland; 2 Department of Biological Bases of Food and Feed Technologies, University of Life Sciences in Lublin, Głęboka 28, 20-612 Lublin, Poland

**Keywords:** Chemical engineering, Green chemistry, Chemical engineering, Mechanical engineering

## Abstract

This paper studies the effect of different ultrasonic parameters on the yield of extraction and antioxidant activity of selected phenolic compounds from *Sorbus intermedia* berries. The sonication was carried out in two modes: continuous and pulse. In the pulse mode, the samples were sonicated with the following processor settings: 1 s on–2 s off. The effective ultrasonic processor times were 5, 10, and 15 min, and the total extraction times were 15, 30, and 45 min. The results showed that the duty cycle significantly affected the antioxidant activity of the extracts and the yield of chlorogenic acid, rutin, and total flavonoids. Compared to the continuous mode, the pulse ultrasound increased the extraction yield of rutin by 5–27%, chlorogenic acid by 12–29%, and total flavonoids by 8–42%. The effect of the duty cycle on the extraction yield was dependent on the intensity and duration of the ultrasound treatment. The mechanism of the influence of the pulsed ultrasound field on the extraction process has been elucidated. This research clearly demonstrated the superiority of pulsed ultrasound-assisted extraction for production of antioxidants from *Sorbus intermedia* berries.

## Introduction

Given the rising costs of energy and the need to reduce greenhouse gas emissions, the food, pharmaceutical, and natural pharmaceutical industries are focused on the use of more efficient and environmentally friendly techniques for extraction of bioactive substances^1^. Ultrasound-Assisted Extraction (UAE) may be one of such methods. Compared to other techniques, this type of separation has many advantages, e.g. higher efficiency, lower process temperature, low consumption of solvents, and relatively low costs of equipment^2^ . Moreover, this economical method shortens the process time and lowers energy consumption^3,4^.

The mechanism of the ultrasound-assisted extraction process consists in intensification of the extraction of bioactive compounds from plant raw materials with the use of high-intensity sound waves. Sonication contributes to partial disintegration of plant tissue through acoustic cavitation and accelerates the release of extracted components into the solvent by increasing the mass transport^5^.

The efficiency of ultrasound-assisted extraction is influenced by the following factors: the ultrasound intensity and frequency, the process time and temperature, the liquid-to-solid ratio, the type, concentration, and pH of the solvent, and the sonicator duty cycle (continuous or pulse)^6,7^.

The UEA efficiency increases with increasing ultrasound intensity and then decreases after reaching a certain critical value specific for a given bioactive substance^8,9^. The higher efficiency of the process is related to increased disintegration of plant tissue and increased solvent penetration related to a rapid collapse of cavitation bubbles. However, high-intensity ultrasound may degrade the bioactive compound, thereby reducing the extraction yield^10^.

Another determinant of the extraction efficiency is the ultrasound frequency. The number of reports on this issue is limited, and most research has been carried out at a constant frequency of ultrasound. Researchers usually use low ultrasound frequencies due to the possibility of induction of the cavitation phenomenon at a lower level of ultrasound intensity^11–16^.

Another important parameter of the extraction process is the selection of an appropriate solvent. Ethanol and its solutions are used most commonly. It has been found that ethyl alcohol has the highest affinity for phenolic compounds in many systems and is therefore the first choice for the extraction of phenolic compounds from plant materials^17^. An increase in the concentration of ethanol in an aqueous solution initially increases the yield of phenolic compound extraction and then reduces the efficiency^10^. The efficiency of ultrasound-assisted extraction also rises with the increasing liquid-to-solid ratio. A greater difference in solute concentrations increases the diffusivity and dissolution of the solute in the solvent, thereby supporting the extraction process. The cavitation intensity at a high liquid-to-solid ratio is higher and causes greater fragmentation, erosion, and pore-formation, thus increasing the extraction efficiency. However, the bioactive substance may undergo degradation after a certain level of cavitation intensity has been exceeded^18,19^.

The number of reports on the impact of solvent pH on the extraction of phenolic compounds is limited, and only Rodrigues et al.^20,21^ determined the optimal solvent pH to be in the range of 1–3.

The temperature is an important determinant of the extraction efficiency. It improves the desorption properties and solubility of the substance dissolved in the solvent. Additionally, it reduces the viscosity of the solvent, which increases the diffusivity of the solvent and the solute. In turn, an excessive temperature rise weakens the cavitation effect^10,22^.

The effect of the ultrasound-assisted extraction time on the content of bioactive compounds has been extensively studied. It is known that a longer sonication time initially increases but then reduces the efficiency. The initial extension of the sonication time accelerates the processes of raw material swelling and hydration as well as fragmentation of plant cells in which the soluble substance is dissolved. An extremely long ultrasonic extraction time results in solute degradation and reduces the extraction efficiency. In the case of the extraction of phenolic compounds, the optimal extraction time has been estimated at 10–90 min, depending on the type of raw material^10–16^.

A less known issue is the effect of the ultrasonic processor duty cycle on the efficiency of extraction of bioactive substances. Pan et al.^23^ reported no significant differences in the extraction yield and duration related to the ultrasound duty cycle, but approximately 50% lower electricity consumption was observed in the pulse mode. Similar results were obtained by Patience et al.^24^ during the extraction of pectins from orange peels. There was no significant difference in the yield between the continuous and pulse modes despite the large difference in power consumption (190 kJ *vs*. 80 kJ).

In contrast, Christou et al.^25^ recovered polyphenols from mature and unripe carob (carob flour) and achieved slightly higher yields with the use of pulse-mode UAE. In turn, Kobus et al.^26^ showed that the use of the pulsed ultrasonic field resulted in a 1.14–34% increase in the yield of extracted polyphenols and anthocyanins in comparison with the continuous ultrasonic field variant. Xu et al.^19^ showed that, compared to CHE (conventional heating extraction), ultrasound-assisted extraction in the pulse mode (2 s on–2 s off) improved the efficiency of the process by 26.74% and shortened the extraction time (51.79 min).

Currently, there is increasing interest in extraction methods and research of bioactive substances contained in less known fruit species due to their potential health benefits in the prevention of chronic diseases^27^. *Sorbus L.* species are widely distributed in the northern hemisphere^28^. *Sorbus intermedia* is native to north-western Europe but now naturally found in other parts of Europe^29^. Extracts of various *Sorbus L.* species are used as a traditional remedy for various digestive disorders, treating respiratory tract infections, flu, fever, cold, rheumatism, anaemia, gout, oedema, dyspepsia^30^. The chemical composition of fruits of various species of rowan is similar^31^. Rowan fruits contain organic acids: sorbic, parasorbic, citric, malic, tartaric and succinic, phenolic acids: vanillin, coffee, gallic, p-coumaric, ellagic, syringic, ferulic, benzoic and protocatechic^31,32^. It has also been found flavonoids: quercetin, kaempferol, isorhamnetin, rutin, isoquercetin and jaceosidin, anthocyanins, vitamins and bioelements^31^. Phytochemical studies on *Sorbus intermedia* are limited. Articles refer to the presence of flavonoids and phenolic acids^33–36^. Sołtys et al.^37^ analyzed of *Sorbus intermedia* fruits with regard to their triterpenoid composition.

The promising results of the research on the extraction of polyphenols and anthocyanins from hawthorn with the ultrasound-assisted method have prompted us to extend the scope of the research to include other raw material and other bioactive substances. Hence, the aim of the study was to compare the effect of the ultrasonic processor duty cycle on the yield of bioactive compounds and the antioxidant activity of the extracts.

## Materials and methods

### Study material

Plant material (fruits) of *Sorbus intermedia (Ehrh.) Pers* was collected in October of 2020 in Lublin Upland in Poland. DSc Wojciech Durlak identified it as *Sorbus intermedia*. The specimen vouchers (*S. intermedia* No. 12/2021) were deposited by DSc Wojciech Durlak in the herbarium of the Horticultural Production Institute, Faculty of Horticulture and Landscape Architecture, University of Life Sciences in Lublin. The collection of plant material and experiment had been conducted in compliance with relevant guidelines and regulations. Immediately after harvesting, the raw material was freeze-dried in a lyophilizer. Next, the material was crushed and divided into 5 fractions. Three fractions were selected for further analyses. They were obtained by sieving through sieves with different mesh diameters: fraction 1—0.25–0.5 mm mesh diam., fraction 2—0.5–1.0 mm mesh diam., and fraction 3—1.0–2.0 mm mesh diam.

### Ultrasonic treatment

Four grams of raw material from each fraction were placed in the extraction cell and covered with an aqueous solution of 60% ethyl alcohol. Next, the extraction cell was placed in a cooling jacket connected to an ultra-thermostat to stabilize the temperature. The extraction cell was closed with a 19-mm diameter. ultrasonic probe on the top. The experimental samples were sonicated with a VC750 Sonics processor (Sonics and Materials Inc., USA) operating at a frequency of 20 kHz. The sonication was performed at three amplitudes of 12, 24, and 36 µm corresponding to ultrasound intensities of 2.5, 9.5, and 19 W/cm^2^, respectively, in the case of continuous mode. In the case of the pulse mode, the mean ultrasound intensities were lower, i.e. 1.3, 7.5, and 14 W/cm^2^ for the amplitudes of 12, 24, and 36 µm, respectively^26^.

The ultrasonic treatment was carried out in continuous and pulse modes. In the pulse mode, the samples were sonicated at the 1 s on–2 s off processor duty cycle. The effective operation times were 5, 10, and 15 min, and the total extraction times were 15 min, 30 min, and 45 min, respectively. In the continuous mode, the samples were sonicated for 5, 10, and 15 min, respectively, and then kept in the extraction cells for 10, 20, and 30 min to achieve the same extraction times as in the pulse mode. The extracts were stored under refrigeration (2 °C) and collected for further chemical analyses^26^.

### Chemical analyses

#### Determination of flavonoid content (TFC)

The content of flavonoids was determined with the spectrophotometric method using quercetin as a reference standard^38^. First, the sample extract (1.0 mL) was mixed with 1 mL of a 2% AlCl_3_ × 6 H_2_O solution (in methanol), and the mixture was made up to 10 mL with distilled water. After incubation of the mixture for 10 min at room temperature in the dark, the absorbance was measured at 430 nm. A calibration curve was prepared with quercetin and the results were expressed as mg quercetin equivalent per 1 g of dry matter (mg QE g^−1^ dry matter).

#### Determination of chlorogenic acid and rutin by HPLC

Chlorogenic acid and rutin in the extracts were quantified using a modified version of the HPLC (High Performance Liquid Chromatography) method proposed by Alberti et al.^39^. The analysis was conducted using HPLC System S 600 Series equipment (Sykam GmbH, Eresing, Germany) coupled to a photodiode arrangement detector (PDA S 3345, Sykam). All extract were filtered through a 0.20 µm syringe filter (Nylon, Macherey–Nagel, Düren, Germany), and 20 µL of the sample was injected in the system in triplicate. The separation was carried out using a Bionacom Velocity STR (3.0 mm × 250 mm, 5.0 µm) column at 25 °C^40^.

The mobile phases consisted of 25 mL·L^−1^ of acetic acid (solvent A) and acetonitrile (solvent B). The system was run with at a flow rate of 1.0 mL·min^−1^ in the following gradient program: 3–9% B (0–5 min), 9–11% B (5–13 min), 13–30% B (13–20 min), 30% B (20–25 min), and 30–3% B (25–27 min). The peaks of the compounds were identified and quantified by comparison of the retention times and spectra of the samples with calibration curves that had been previously prepared with standards. The runs were monitored at 260 nm^40^.

#### Determination of antioxidant activity

##### Determination of free radical scavenging activity with the DPPH assay

The antioxidant activity of the extracts was evaluated using the DPPH (2,2-diphenyl-1-picrylhydrazyl) assay^41^. For this analysis, 0.2 mL of the extract was mixed with an aliquot of 5.8 mL of freshly prepared 6·10^−5^ M DPPH radical in methanol. The mixture was allowed to stand at room temperature for 30 min, and the spectrophotometric absorbance was measured at 516 nm using methanol as a blank. ^40^ The measurement was performed in three replicates for each sample. Antioxidant activity was expressed as a Trolox equivalent in µg per g of dry matter.

##### Ferric reducing antioxidant power assay (FRAP)

The ferric reducing antioxidant power of the extract was evaluated according to the method described by Benzie & Strain^42^ with some modifications. First, the working reagent was prepared as a mixture of 20 mM FeCl_3_, 300 mM acetic acid, and 10 mM of TPTZ in 10:1:1 (v/v/v) proportion in 40 mMHCl. Next, 30-μL of the extracts were mixed with 5 mL of the FRAP solution. After incubation at 37 °C for 10 min, absorbance of the mixture was measured at 593 nm. The FRPA values of the extracts were expressed as a Trolox equivalent in µg per g of dry matter.

### Statistical analyses

The results were statistically analyzed with Statistica software via analysis of variance (ANOVA). The significance of differences between the evaluated mean values (in figures) was analyzed with the Tukey test at a significance level of *p* < 0.05^26^**.**

The tables present the mean values with standard deviations, while the graphs present the mean values and whiskers representing standard deviations. The results of the chemical properties are presented as average values of three measurements in each sample.

## Results

### Flavonoids

**Figure 1:**
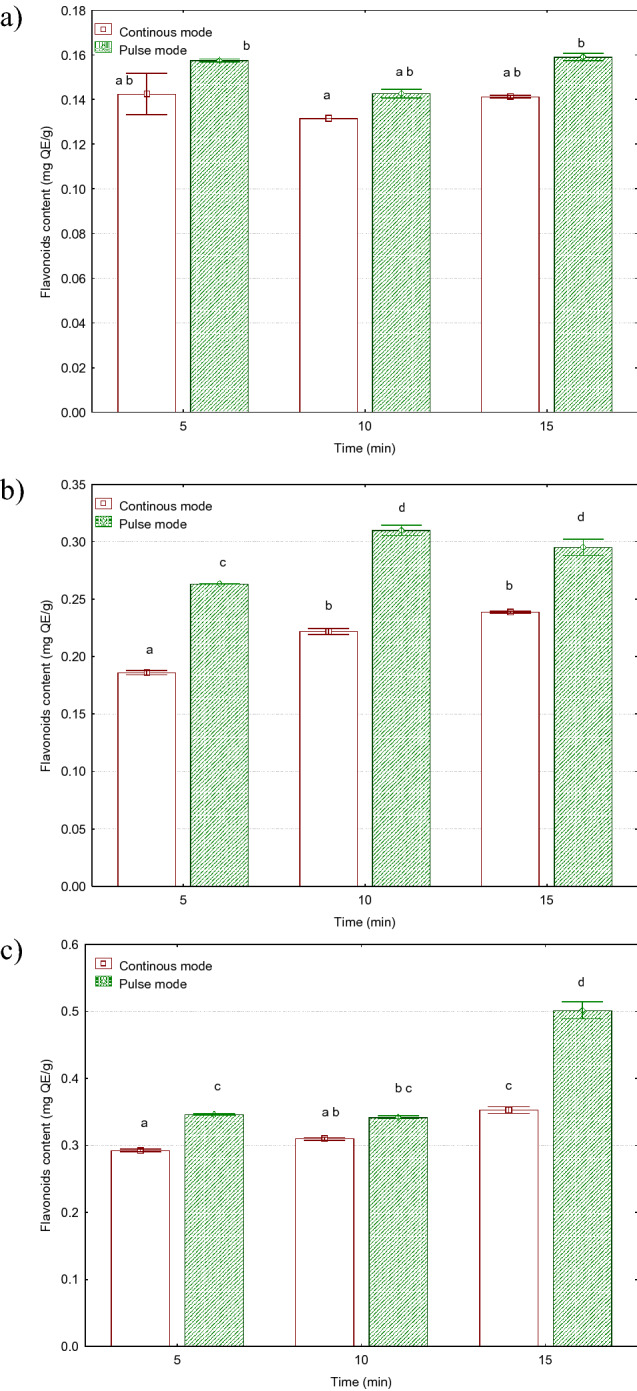
Effect of ultrasonic treatment parameters on the content of flavonoids (mg QE/g d.m.) in *Sorbus intermedia* berries extracts: (**a**) 12 µm, (**b**) 24 µm (**c**) 36 µm.

Flavonoids are bioactive compounds with multiple health-enhancing properties, e.g. antioxidant, anti-inflammatory, anti-cancer, and detoxifying activity. Figure 1 shows the effect of the type of treatment (continuous, pulse) on the content of flavonoids in *Sorbus intermedia* alcoholic extracts. The highest content of flavonoids, i.e. 0.502 mg QE/g, was detected in extracts obtained in the pulse mode at the 36 µm amplitude and 15 min treatment time. The lowest amounts of flavonoids, i.e. 0.132 mg QE/g, were extracted in the continuous mode (12 µm amplitude and 5-min time). The increase in the pulse amplitude from 12 µm to 36 µm resulted in an increase in the flavonoid content from 105 to 216%. Another important determinant of the yield was the extraction time. The differences in the extraction between the shortest and longest extraction time ranged from 12 to 45%. The duty cycle of the ultrasonic processor also had a positive effect on the content of flavonoids. The difference in the flavonoid content between the pulse and continuous mode variants ranged from 8 to 42%. In the case of the 12 µm amplitude, the processor duty cycle did not exert a statistically significant effect on the content of flavonoids, whereas the differences in the case of the 24 and 36 µm amplitudes turned out to be statistically significant.

### The antioxidant activity of *S. intermedia* berry extracts was assessed using the FRAP and DPPH methods

#### DPPH

Table 1 shows the effect of the sonication parameters on the antioxidant activity of *Sorbus intermedia* extracts determined with the DPPH method. The highest values were obtained in the case of the pulse mode with the following processing parameters: 24 µm amplitude and 15 min time. The lowest values were noted in the continuous mode with 12 µm amplitude and 5 min time. The difference in between the variants with these processing parameters was 129%. The extracts obtained with the use of the pulsed ultrasound field exhibited higher antioxidant activity than the extracts from the continuous mode treatment. These differences turned out to be statistically significant for the 12 µm amplitude and 15 min time and for the 24 µm amplitude and the time of 5, 10, and 15 min. The greatest impact on the antioxidant activity was exerted by the ultrasonic vibration amplitude. The increase in the vibration amplitude from 12 µm to 36 µm resulted in an increase in the DPPH value from 66 to 120%. Another important parameter was the ultrasonic treatment time. An increase in the treatment time from 5 to 15 min resulted in an increase in the DPPH value from 1.5% to 37%.

**Table 1:** DPPH values in individual extracts.

Amplitude [µm]	Time [min]	Pulse mode	Continuous mode
		DPPH [µM TE/1 g]	
12	5	42.745 ± 0.598 a A	32.789 ± 2.598 a A
	10	41.611 ± 0.412 a A	34.404 ± 1.209 a A
	15	44.892 ± 2.874 a A	44.974 ± 2.123 b A
24	5	60.906 ± 1.822 a A	51.390 ± 0.181 b A
	10	73.485 ± 0.301 a B	66.709 ± 0.395 b B
	15	75.000 ± 0.591 a B	66.183 ± 0.704 b B
36	5	70.985 ± 0.378 a A	63.346 ± 2.044 a A
	10	74.228 ± 0.892 a A	68.979 ± 1.975 a A
	15	72.044 ± 4.637 a A	74.384 ± 0.446 a A

a, b—different letters show statistically significant differences in Tukey’s test in row (< 0.05).

A, B—different letters show statistically significant differences in Tukey’s test in column for one amplitude (< 0.05).

#### FRAP

Table 2 shows the effect of the treatment type (continuous, pulse) on the antioxidant activity of the *Sorbus intermedia* extracts determined with the FRAP method. The highest antioxidant activity was detected in extracts obtained in the pulse-mode treatment with the following parameters: 36 µm amplitude and 15 min time. The lowest values were noted in the continuous-mode treatment with the 12 µm amplitude and 5 min time. The difference between the extreme values reached 287%. The amplitude had the greatest effect on the ability to scavenge free radicals. The increase in the vibration amplitude from 12 µm to 36 µm resulted in an increase in the FRAP value from 96 to 196%. Another important determinant of the antioxidant capacity in the *Sorbus intermedia* extracts was the extraction time. The increase in the sonication time from 5 to 15 min resulted in an increase in the FRAP value from 12 to 48%. The difference in the FRAP values between the pulse and continuous modes ranged from 11 to 26%. The processor duty cycle was found to exert a statistically significant effect on the reducing power.

**Table 2:** FRAP values in individual extracts.

Amplitude [µm]	Time [min]	Pulse mode	Continuous mode
		FRAP [µM TE/1 g]	
12	5	16.802 ± 0.232 a A	13.065 ± 0.187 b A
	10	15.938 ± 0.175 a A	11.720 ± 0.282 b A
	15	16.278 ± 0.476 a A	18.700 ± 0.228 b B
24	5	29.287 ± 0.415 a A	22.965 ± 0.063 b A
	10	43.613 ± 0.015 a B	34.279 ± 0.530 b B
	15	43.095 ± 1.623 a B	34.073 ± 0.176 b B
36	5	36.420 ± 1.561 a A	32.595 ± 0.939 a A
	10	42.924 ± 0.072 a B	34.663 ± 0.495 b A
	15	45.312 ± 1.561 a B	36.584 ± 0.983 b A

a, b—different letters show statistically significant differences in Tukey’s test in row (< 0.05).

A, B—different letters show statistically significant differences in Tukey’s test in column for one amplitude (< 0.05).

### Chromatographic analysis

#### Chlorogenic acid

**Figure 2:**
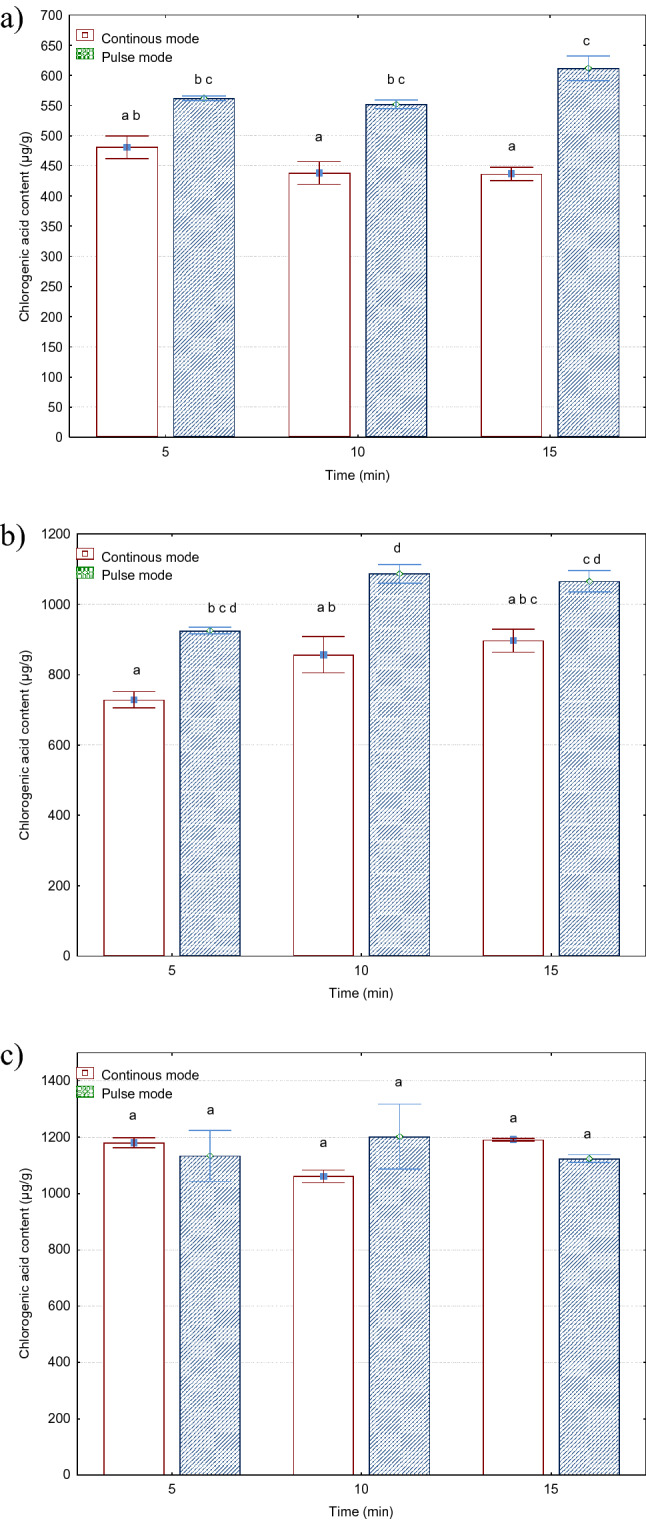
Effect of ultrasonic treatment parameters on the content of chlorogenic acid (µg/g d.m.) in *Sorbus intermedia* berries extracts: (**a**) amplitude 12 µm, (**b**) amplitude 24 µm, (**c**) amplitude 36 µm.

Chlorogenic acid is a phenolic compound from the hydroxycinnamic acid family and it is one of the most common compounds among the polyphenol group contained in *Sorbus intermedia* raw material. Chlorogenic acid possesses many health-promoting properties, most of them related to the treatment of metabolic syndrome, including anti-oxidant, antilipidemic, antidiabetic, anti-inflammatory, and antihypertensive activities. This polyphenol has shown antimicrobial activity against a wide range of organisms^43^. Figure 2 shows the effect of the treatment mode (continuous, pulse) on the content of chlorogenic acid in the *Sorbus intermedia* alcoholic extracts. In the case of the 12 and 24 µm amplitudes, there was a statistically significant effect of the processor duty cycle on the chlorogenic acid content. The highest values were found in the case of the pulse-mode treatment with the following processing parameters: 36 µm amplitude and 10 min time. The lowest values were obtained in the continuous-mode sonication at the 12 µm amplitude and 15 min time. The difference between these values was estimated at 176%. The ultrasonic vibration amplitude exerted the greatest impact on the chlorogenic acid content. The increase in the vibration amplitude from 12 µm to 36 µm resulted in an increase in the chlorogenic acid content from 84 to 146%. The increase in the treatment time from 5 to 15 min contributed to an increase in the chlorogenic acid content in the range from 1 to 23%. The differences in the content of this acid between the variants with the different processor duty cycles ranged from 12 to 29%.

#### Rutin

**Figure 3:**
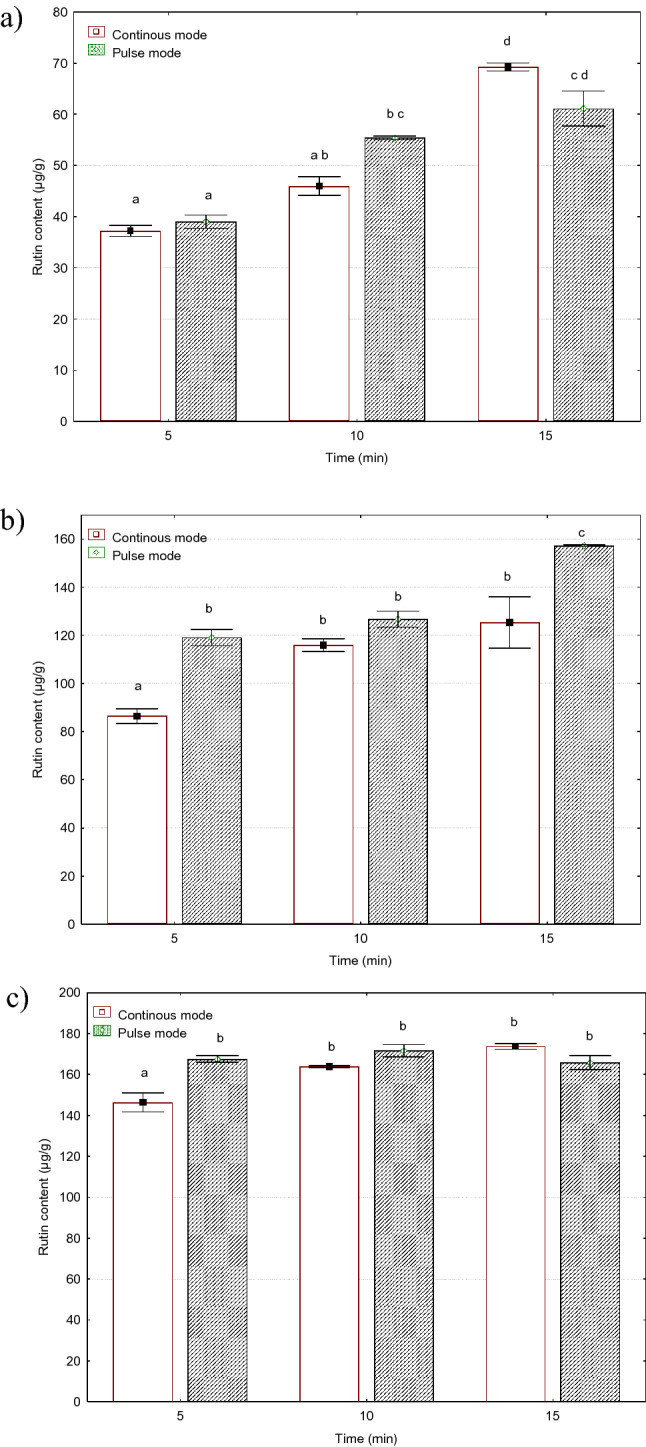
Effect of ultrasonic treatment parameters on the content of rutin (µg/g d.m.) in *Sorbus intermedia* berries extracts: (**a**) amplitude 12 µm, (**b**) amplitude 24 µm, (**c**) amplitude 36 µm.

Rutin is an organic chemical compound from the group of flavonoid glycosides. Rutin demonstrated a lot of medical activities, including anticarcinogenic, antioxidant, chemotherapeutic, cytoprotective, neuroprotective, vasoprotective, and cardioprotective. Rutin has an effect on the prevention of neuroinflammation, promotion of neural crest cell survival, sedative activity, anticonvulsant activity, anti- Alzheimer activity, and treatment of hyperkinetic movement disorder, antidepressant, analgesic and antinociceptive, antiarthritic, antidiabetic, anti-hypercholesterolemic, antiplatelet aggregatory, antiulcer antiasthmatic activity and other associated, antiosteoporotic and antiosteopenic, anticataract and ophthalmic, diuretic, anticancer^44^. Figure 3 shows the effect of the treatment mode (continuous, pulse) on the content of rutin in the *Sorbus intermedia* alcoholic extracts. At the amplitudes of 12 and 24 µm, there were statistically significant differences between extracts obtained in the pulse-mode treatment and those from the continuous-mode process. Higher levels of rutin were observed in the case of the pulse mode. The difference in the rutin content between the pulse and continuous modes ranged from 5 to 27%. The highest values were found for the continuous-mode treatment at the 36 µm amplitude and 15 min time, and the lowest values were obtained at the 12 µm amplitude and 5 min time. The difference between these values reached 367%. The increase in the vibration amplitude from 12 µm to 36 µm resulted in an increase in the rutin content from 150 to 329%. The growth in the treatment time from 5 to 15 min contributed to a rise in the rutin content from 19 to 86%.

## Discussion

The content of bioactive compounds is strongly determined by the genotype and habitat conditions. This indicates that, in addition to the species, such factors as plant height, light, temperature, content of nutrients available in the soil, the sampling site, and the harvesting time (maturity stage) play an important role^45^. Moreover, the content of bioactive compounds depends on the extraction method.

There are no literature reports on the analysis of the TFC content in *Sorbus intermedia*. The number of available literature reports on the content of chlorogenic acids, rutin and values of DPPH and FRAP in *Sorbus intermedia* is limited, and the comparison of the present results poses many problems. Therefore, the discussion refers to other *Sorbus* species.

The values of TFC obtained in the present experiment ranged from 0.132 to 0.502 mg QE/g DM. Jin et al.^46^ evaluated the chemical composition of *Sorbus commixta* fruit extracts prepared with different ethanol concentrations. The total flavonoid content ranged from 2.49 to 8.65 μg QE/mg of extract. Turumtay et al. ^32^ showed that the values of TFC for *Sorbus aucuparia* was 12.336 mg QE/g dried extract and for endemic *Sorbus caucasica var. yaltirikii* was 17.128 mg QE/g dried extract. Majić et al. ^47^ assess service tree (*Sorbus domestica L.*) bark, fruit exocarp and mesocarp, and seeds. The value of TFC ranged from 6.8 to 37.0 mg QE/g DM.

Several methods have been developed to determine antioxidant activity of plant materials. In our study, two methods (DPPH and FRAP) were used to evaluate the antioxidant potential of *Sorbus intermedia* fruit. FRAP is based on ferric reducing power and DPPH is based on the ability to scavenge DPPH radical. The antioxidant activity of the extracts analyzed in the present study determined with the DPPH method ranged from 32.79 to 74.38 µmol TE/g DM, while the extracts determined with the FRAP method ranged from 11.72 to 45.31 µmol TE/g DM. There are very little data in the literature with which our results could be easily compared, given the methods of extraction, analysis, units and plant species. Mrkonjić et al. ^48^ determined DPPH and FRAP values in methanolic extracts from the fruits of *Sorbus intermedia*, but they express their results in ascorbic acid equivalents. They obtained the following data: DPPH 0.20 ± 0.01 mg of AAEc/g DW and FRAP 4.47 ± 0.39 mg of AAEc/g DW. Olszewska and Michel^36^ evaluated the antioxidant potential of 70% methanolic extracts from the fruits of *Sorbus aucuparia*, *Sorbus aria* and *Sorbus intermedia*. According to their research the fruits of *Sorbus intermedia* showed antiradical efficiency of 86.9 µmol TE/g DM for DPPH method and 221.1 µmol TE/g DM for FRAP method. The results of the present experiment show that the levels of DPPH values coincide with the values reported by Olszewska and Michel^36^, but FRAP values are much lower. Probably the main reason for the differences in antioxidant activity determined by the FRAP method was the different incubation time of the samples. Both Olszewska and Mitchel^36^ and Stratil et al.^49^ proved that DPPH and FRAP tests are strongly depended on incubation time and the reactivity of various phenolic standards and plant extracts. Antioxidant activity of extracts are also related with the polarity of solvent. Bobinaite et al.^50^ demonstrated that antioxidant capacity of *Sorbus aucuparia* for water extracts in DPPH and FRAP system were 309 µmol TE/g and 323 µmol TE/g, while ethanol extracts were 103 µmol TE/g and 118 µg TE/g, respectively.

The results of the present experiment show that the levels of chlorogenic acid ranged from 436.43 µg/g DM to 1202.66 µg/g DM. Olszewska and Michel^36^ evaluated the content of chlorogenic acid isomers quantified by HPLC in 70% methanolic extracts from the fruits of *Sorbus intermedia* and the value of chlorogenic acid was 0.23 ± 0.01%. Šavikin et al.^51^ showed that the levels of chlorogenic acid in *Sorbus aucuparia* extracts ranged from 0.35 to 10.01 mg/g DW, while in *Sorbus aria* ranged from 0.22 to 2.30 mg/g DW. Kylli et al. ^52^ indicated that the value of chlorogenic acid in wild rowanberries (*Sorbus aucuparia*) was 5.36 ± 0.10 mg/g D. Mrkonjić et al.^53^ determined chlorogenic acid in water and methanol extracts, and in the jam of *Sorbus aucuparia*. The chlorogenic acid content was were 5.69, 5.80 and 2.60 mg/g DW, respectively. Bobinaitė et al.^50^ evaluated acetone, ethanol and water extracts of rowanberry (*Sorbus aucuparia L.*) pomace. The value of chlorogenic acid was the highest in ethanol extract (3970 µg/g extract). Jin et al.^46^ showed that the concentration of chlorogenic acid ranged from 111.81 to 344.7 µg/g extract.

Olszewska^35^ showed that rutin and isoquercitrin were predominant components of the extracts. In the present study, the rutin levels ranged from 37.22 to 173.76 µg/g DM. The concentrations of rutin in water and methanol extracts of *Sorbus aucuparia* were 82.3 and 80.4 mg/g DW, respectively^48^. Šavikin et al.^51^ indicated that rutin content ranged from 138.4 to 892.0 μg/g DW in *Sorbus aria* and 40.1 to 598.3 μg/g DW in *Sorbus aucuparia* extracts. Bobinaitė et al.^50^ showed that the value of rutin in ethanol extract was 123.9 µg/g extract. Rutin content in ethanolic extracts of *Sorbus commixta* ranged from 0.28 to 0.6 µg/g extract^46^.

The growing interest in green chemistry and the possibility of using ultrasounds have contributed to the increasing application of this method in the extraction of bioactive compounds. Ultrasounds increase mass transfer and contribute to disintegration of plant tissues and cells, which results in a shorter extraction time and higher yields. The available literature provides many examples of the application of ultrasounds to intensify the polyphenol extraction process. Phenolic compounds and antioxidant compounds have been extracted from mango peel^12^, coconut shell powder^20^, pomegranate peel^23^, jabuticaba peel^21^, grape by-product^16^, black chokeberry by-products^17^ and used coffee grounds^22^.

Pulsed ultrasonic field is increasingly being used to support the extraction process. However, the number of studies focused on the direct comparison of the continuous and pulse ultrasonic processor modes in the extraction of phenolic compounds is highly limited. Pan et al.^23^ found no significant differences between the processor duty cycles during extraction of polyphenols from pomegranate peel. However, the pulsed ultrasonic field was found to save approximately 50% of energy in comparison with the continuous sonication mode.

Kobus et al.^26^ demonstrated a statistically significant effect of the ultrasonic processor duty cycle on the extraction of bioactive substances. The increase in the yield of extracted total polyphenols and anthocyanins ranged from 1.14% to 34% in the case of the pulse mode. The study also showed a reduction in energy consumption from 20 to 51% in the process of extraction assisted with the pulsed ultrasonic field. As reported by Ravanfar et al.^54^, this mode (1 s on–1 s off) had an approx. 1.5-fold higher efficiency than the continuous mode in the case of anthocyanin extraction.

There are also ambiguous reports on the impact of the duty cycle length on the efficiency of pulsed field-assisted extraction. Xu et al.^19^ observed an increase in the yield of pectin extraction from grapefruit peel along with the increase in the pulse duration/pulse interval ratio from 30 to 50% followed by a decrease after exceeding this threshold. More and Arya^55^ reported a significant effect of the interactions between the ultrasonic power and the duty cycle on the efficiency of extraction of polyphenolic compounds from pomegranate peel. Maximum extraction efficiency was achieved with an 80% duty cycle. A similar statistically significant effect of the duty cycle on the yield was obtained by Li et al.^56^ during extraction of curcuminoids from dried turmeric and by Kazemi^57^ during extraction of phenolics from pomegranate peel. Jain et al.^58^ studied the effect of various parameters such as extraction time (min), ultrasonic amplitude (%) and pulse interval (s) with 2 M concentration of hydrotropes and showed that the highest composition of patchoulol i.e. 70.06% was obtained at a 5-min extraction time, 40% ultrasonic amplitude, and a 30:30 s pulse interval. Rakshit et al.^59^ showed that an increase in the duty cycle from 60 to 90% was accompanied by an increase in the concentration of punicalagin (98–152 mg/g). A study conducted by Kaderides et al.^60^ showed that the combination of 10-s intervals with 10-s pauses in the ultrasonic processor work was the best scheme for extraction of polyphenols from pomegranate peel and ensured low energy consumption. The authors demonstrated that the ultrasonic pulse/pause duration ratio of 1.2/1 should be applied in the process of pomegranate peel extraction. Appropriate selection of the pulse duration and intervals between pulses is highly important for reduction of the total extraction time and energy consumption. The extraction yields increased with the decreasing value of the pulse duration/pause ratio^61^. A further reduction in this ratio may cause degradation of plant material^60^. This indicates that an adequate use of the duty cycle may have a beneficial effect on the extraction efficiency. In contrast, Luque-Garcia and De Castro^62^ reported that the duty cycle exerted an insignificant effect on fat recovery from oleaginous seeds.

In the present study, there was a positive and statistically significant effect of the ultrasonic processor duty cycle on the extraction efficiency of all substances, i.e. total flavonoids, rutin, and chlorogenic acid, and on the antioxidant activity of the extracts determined with the FRAP and DPPH methods. The increase in the extraction yield in the pulse mode variant ranged from 8 to 42% for TFC, from 11 to 26% for FRAP, from 7 to 23% for DPPH, from 12 to 29% for chlorogenic acid, and from 5 to 27% for rutin and depended on the amplitude of ultrasonic vibrations and the processing time.

The higher extraction efficiency can be explained by the slight differences in the mechanism of the pulsed acoustic field compared to the continuous field. The short duration of ultrasonic pulses results in the formation of fewer cavitation bubbles than in the case of the continuous field treatment. The collapse of such bubbles generates a stronger shock wave, which results in greater fragmentation of plant tissue. The pause following the collapse of the cavitation bubbles allows soluble substances to diffuse into the surrounding solvent. The implosion of cavitation bubbles in the subsequent duty cycle of the ultrasonic processor induces defragmentation of further parts of the plant tissue and contributes to release of another portion of soluble substances. This mode of interaction causes gradual destruction of plant tissue and a lower concentration of the soluble substance in the boundary layer than in the case of the continuous field treatment. Additionally, the smaller number of cavitation bubbles at the surface of the ultrasonic probe reduces the likelihood of the so-called the saturation effect, which reduces the transmission of acoustic energy to the solvent surrounding the probe. The lower concentration of the soluble substance within the solid matrix accelerates the diffusion of the extracted component into the surrounding solvent.

The extraction temperature is an important parameter that may be responsible for the differences in the efficiency of the ultrasonic processor duty cycle. The increase in temperature results in an increase in the yield on the one hand but substantially reduces the cavitation effect of ultrasounds on the other hand. This has been confirmed in many studies^63,64^.

It should also be noted that despite the use of a temperature stabilizing system directly under the ultrasonic probe, the solvent temperature in the continuous mode may temporarily reach considerably higher values than in the case of the pulse mode. In the pulse-mode treatment, the cooling system has substantially more time to dissipate successively generated portions of heat from the surroundings of the ultrasonic probe. The increasing temperature under the ultrasonic probe may be another element that reduces the efficiency of extraction in the continuous field mode.

Summing up, taking into account the type of the processor duty cycle, at least the same or higher extraction yield of each bioactive substance was achieved using the pulse mode. The phenomenon of the different effects of the processor duty cycle on the efficiency of extraction of bioactive components from *Sorbus intermedia* requires further research.

## Conclusions

The present study has shown that the efficiency of the extraction process is strongly determined by the ultrasonic processor duty cycle. This analysis is the first that compares the two operating modes of an ultrasonic processor during extraction of polyphenols from *Sorbus intermedia* berries.

A higher statistically significant value of the process efficiency was obtained in the pulse-mode variant. The largest difference, i.e. in the range from 8 to 42%, was observed during the extraction of total flavonoids analyzed by spectrophotometry. Smaller differences were found for individual polyphenols analyzed with the chromatographic method. In the case of chlorogenic acid, the differences were from 12 to 29% and in the case of rutin from 5 to 27%. The effect of the processor operating mode on individual polyphenolic compounds depended on the amplitude of ultrasonic vibrations.

The higher content of the extracted bioactive compounds was also confirmed by their higher antioxidant activity. The research method was found to exert a significant effect on the antioxidant activity of the extracts. Higher antioxidant activity was revealed by the determinations conducted with the use of the DPPH reagent.
